# Role of γδ T cells in controlling viral infections with a focus on influenza virus: implications for designing novel therapeutic approaches

**DOI:** 10.1186/s12985-020-01449-0

**Published:** 2020-11-12

**Authors:** Ailar Sabbaghi, Seyed Mohammad Miri, Mohsen Keshavarz, Mehran Mahooti, Arghavan Zebardast, Amir Ghaemi

**Affiliations:** 1 Department of Influenza and Other Respiratory Viruses, Pasteur Institute of Iran, P.O. Box 1316943551, Tehran, Iran; 2 The Persian Gulf Tropical Medicine Research Center, The Persian Gulf Biomedical Sciences Research Institute, Bushehr University of Medical Sciences, Bushehr, Iran; 3 Department of Virology, School of Public Health, Tehran University of Medical Sciences, Tehran, Iran

**Keywords:** Influenza virus, Innate immunity, Gamma-delta T cells, Adaptive immunity, Vaccine

## Abstract

**Background:**

Influenza virus infection is among the most detrimental threats to the health of humans and some animals, infecting millions of people annually all around the world and in many thousands of cases giving rise to pneumonia and death. All those health crises happen despite previous and recent developments in anti-influenza vaccination, suggesting the need for employing more sophisticated methods to control this malign infection.

Main body

The innate immunity modules are at the forefront of combating against influenza infection in the respiratory tract, among which, innate T cells, particularly gamma-delta (γδ) T cells, play a critical role in filling the gap needed for adaptive immune cells maturation, linking the innate and adaptive immunity together. Upon infection with influenza virus, production of cytokines and chemokines including CCL3, CCL4, and CCL5 from respiratory epithelium recruits γδ T cells at the site of infection in a CCR5 receptor-dependent fashion. Next, γδ T cells become activated in response to influenza virus infection and produce large amounts of proinflammatory cytokines, especially IL-17A. Regardless of γδ T cells’ roles in triggering the adaptive arm of the immune system, they also protect the respiratory epithelium by cytolytic and non-cytolytic antiviral mechanisms, as well as by enhancing neutrophils and natural killer cells recruitment to the infection site.

**Conclusion:**

In this review, we explored varied strategies of γδ T cells in defense to influenza virus infection and how they can potentially provide balanced protective immune responses against infected cells. The results may provide a potential window for the incorporation of intact or engineered γδ T cells for developing novel antiviral approaches or for immunotherapeutic purposes.

## Background

It has been fully demonstrated that influenza virus infection is a serious menacing threat to human health, causing over 50,000 death tolls around the world annually [1]. In addition, reassortment of influenza virus genome due to antigenic drift and shift leads to pandemic outbreak, resulting in severe diseases such as that observed in the 1918 pandemic strain with over 50 million deaths during 2 years [2]. Although annual vaccination is substantially considered as an effective strategy against influenza virus infection, poor adherence and low efficacy of employed influenza vaccine strains increase the demands for better understanding of host immune responses [3, 4].

Upon influenza virus infection within the cells, the innate compartment of immune system is activated in response to detection of pathogen-associated molecular patterns (PAMPs), specific of viral infection, by pattern recognition receptors (PRRs). Of note, the innate immune system employs three distinct groups of PRRs for recognition of influenza virus including RLRs (retinoic acid-inducible gene-I-like receptors or RIG-I-like receptors), NLRs (NOD-like receptors), and TLRs (toll-like receptors) [5]. This recognition results in the production of different cytokines and chemokines such as interferons (IFNs), which limits the early viral spread and further mediates adaptive immune response [3, 6]. Activated innate response bestows the first line of cellular defense to protect hosts against influenza virus infection. Each leukocyte such as neutrophils, monocytes, natural killer (NK) cells, innate lymphoid cells (ILCs), and gamma-delta (γδ) T cells may contribute to anti-influenza protection through varied strategies.

γδ T cells, which based on the usage of either Vδ1 or Vδ2 domains are classified into two major subgroups [7, 8], are important components of both innate and adaptive immunity, however, they just constitute approximately 1–10% of peripheral blood T cells in humans. It has been established that γδ T cells not only exert anti-influenza activity by direct elimination of virus [9, 10], but also inhibit virus replication through the secretion of IFN-γ [11, 12]. Moreover, a pre-clinical study has shown that the proportion of γδ T cells was around 15–30% of the lymphocytes in the BAL between 10 to 15 days following infection, suggesting the possible role of this type of cell in recovery from influenza infection [13]. Besides, a study demonstrated that γδ T cell is required for initiation of effective immune responses and maintenance of lung homeostasis in influenza-infected individuals [14]. It has been regarded that upon encountering non-peptide phosphoantigens, isopentenyl pyrophosphate (IPP) or pathogen-associated antigens independent from major histocompatibility complex (MHC) presentation, γδ T cells become activated, eventually resulting in regulation of immune responses [15, 16]. It is well established that γδ T cells are predominantly considered as innate immune cells but can develop memory-like adaptive responses, bridging the gap between acquired and innate immunity [17], therefore play pivotal roles in protection against virus infection and in anti-tumor immunity [18, 19].

The aim of this review is to present a framework for the role of γδ T cells in modulating immune responses against various pathogens, particularly influenza virus, and also to describe their applicability to develop novel therapeutic approaches for viral infections as well as their restrictions.

## Main text

### Overview of immune response to influenza infection

Innate cells like neutrophils and macrophages are the first leukocytes infiltrated to the lung upon influenza virus infection and secrete different cytokines, in particular, pro-inflammatory cytokines, causing recruitment of other leukocytes to the infection site [20]. In addition, neutrophil has been shown to present viral antigen on its surface, leading to activation and direction of CD8^+^ T cells into the influenza infected-lung [21]. Moreover, activated macrophage is able to release several inflammatory cytokines that are capable of influenza virus inhibition [22].

Following influenza infection, natural killer cells are recruited into the respiratory tract, mainly through blood, in response to the expression of their chemokine receptors including CCR2, CCR5, and CXCR3 [23–25]. NK cells activation contributes to destruction of influenza-infected cells via secretion of perforins, granzyme B, and IFN-γ [26, 27]. Furthermore, in the early stages of influenza virus infection, innate lymphoid cells including cytotoxic NK cells and other innate lymphoid cells (ILCs), innate counterparts of T cells, have been shown to be effective. In particular, ILC1 induces the cytolysis of influenza-infected cells and ILC2 is trafficked into lung upon influenza infection and becomes activated, releasing amphiregulin (Areg), which contributes to repairing the damaged tissue [28, 29].

Another pivotal member of innate cells is dendritic cell (DC), a professional antigen-presenting cell (APC), bridging innate and adaptive compartments of immune systems. DCs are generally classified into 2 categories: CD11c^+^ conventional DCs (cDCs), and CD11c^−^ plasmacytoid DCs (pDCs) [30, 31]. Compared to these subtypes of DCs, there are CD103^+^ cDCs with the ability to uptake viral antigen and migrate to draining lymph nodes (DLNs) and are known to be the most efficient cross-presenters of the immune system [32, 33]. And these cDCs have been shown to process and present influenza A virus (IAV) antigens to CD8^+^ T cells. Furthermore, both conventional and plasmacytoid DCs act as anti-influenza agents [34].

Recently, more studies have focused on other members of innate immunity including mucosal-associated invariant T (MAIT), natural killer T (NKT), and γδ T cells that respond to the influenza virus.

MAIT cells are evolutionarily-conserved, innate-like lymphocytes constituting 5% of the total T cell pool in humans. MAIT cells express a semi-invariant αβ T cell receptor (TCR), which has been demonstrated to identify bacterial-derived vitamin B2 derivatives presented by the MHC class I-related gene protein (MR1) [35, 36]. Moreover, it has been indicated that MAIT cells contribute to protection against several intracellular bacteria [37, 38]. Accumulating evidence substantiates that a loss and/or efflux of MAIT cells from the blood to the site of infection are observed in patients infected with the influenza virus [39, 40]. These studies manifest that a decrease in the number of MAIT cells during acute influenza infection could impair protective anti-bacterial immunity, leading to the elevation of bacterial co-infection risk that ultimately would result in higher morbidity and mortality [41].

Another innate T cell subset is the natural killer T that is restricted by the glycolipid α-galactosylceramide (αGC) presented on MHC class I-like CD1d molecules. In addition to expressing αβ-TCRs, this subset expresses NK cell markers including CD3, CD56 (humans) or NK1.1 (mice), and is broadly classified into 2 groups: type I invariant NKT (iNKT) cells expressing the invariant Vα14-Jα18 TCRα chain paired with either Vβ2, Vβ7, or Vβ8 in mice and Vα24-Jα18/Vβ11 in humans, and type II diverse NKT cells, displaying very diverse αβ-TCR gene pairings [42, 43]. An array of animal studies demonstrated that activation of iNKT cells leads to a reduction of body weight loss and viral lung titers [44, 45]. Similarly, one study showed that the αGC usage as a vaccine adjuvant improved the long-term survival of CD8^+^ cytotoxic T lymphocytes, providing heterologous protection in a mouse model of influenza infection [46]. Intriguingly, one pre-clinical study demonstrated that vaccination with inactivated influenza A/PR/8/34 virus adjuvanted with αGC in BALB/c mice elicited IgG and IgA antibodies, which lasted for months and further boosted cellular immunity in comparison to mice immunized just with inactivated PR8 virus [47]. Taking together, this subset of innate T cell is considered as an innate and adaptive immune response modulator based on its role in establishing optimal influenza-specific T and B cell responses in the mucosal and systemic compartments. Another important participant in the immune response against influenza infection is the γδ T cell, which we will talk about later.

On the other hand, there is an adaptive immune response that differs from innate immunity. The adaptive immune system-belonging cells present various receptors with precise specificity for antigens derived from particular pathogens. This adaptive immunity constitutes the second line of barrier against influenza virus infection. The adaptive-attributed humoral and cellular immune responses are mediated by virus-specific antibodies and T cells, respectively [48]. Following influenza infection, virus-specific antibodies are elicited and those specific for surface glycoproteins, hemagglutinin (HA) and neuraminidase (NA), are directed to these antigens, resulting in virus neutralization and spread limitation, respectively [49, 50]. Of note, this antibody-mediated immune response is predominantly strain-specific [51].

Activation of different immune cells upon influenza infection further induces cellular immunity consisting of CD4^+^ T cells, CD8^+^ T cells, and regulatory T cells (Tregs). Following recognition of virus-derived MHC class II-associated peptides on APCs by CD4^+^ T cell, this T cell becomes activated and forms either T-helper 1 (Th1) or T-helper 2 (Th2) type T cells [51]. Th2 cells produce IL-4 and IL-13, leading to the promotion of B cell responses [52], whereas, Th1 cells produce IFN-γ and IL-2 that mainly contribute to cellular immune responses [53]. In addition, virus-specific CD8^+^ T cells, as mentioned before, are recruited at the site of infection and are able to recognize and eliminate influenza virus-infected cells and therefore prevent production of progeny virus [48].

### Principles of γδ T cells

T lymphocytes are classified structurally into two subgroups based on different glycoprotein chains of T cell receptors known as αβ and γδ, each of which are composed of a variable (V), constant (C), and joining (J) segment generated through VDJ recombination [54, 55]. The γδ-TCR loci gene rearrangement occurs at an early stage in the fetal thymus and prior to αβ-TCR genes rearrangement [56], therefore, intraepithelial lymphocytes (IELs) with γδ-TCR (γδ IELs) are believed to be the initial type of T cells localizing in the epithelium and so play an important role in the immune responses during the perinatal period, prior to TCR αβ T cell activation [57].

Among two major subgroups of γδ T cells, defined by the usage of either Vδ1 or Vδ2 TCR, Vδ1 subset, paired with different Vγ chains (comprising Vγ2/3/4/5/8), constitutes the less frequent population and is mainly found in epithelial tissues and mucosae and involves in the first barrier defense against infections, whereas Vδ2 subset, which is uniquely associated with Vγ9 chain (called Vγ9 Vδ2), is more abundant in peripheral blood and plays roles in defense against intracellular and circulating pathogens [58, 59]. By contrast, γδ T cells expressing Vδ3 with either Vγ2 or Vγ3 (termed Vδ3 subset) are the smallest subset of γδ T cells predominantly resident in the liver and take participate in the process of chronic viral infections. Furthermore, the functionality categories of γδ T cells, e.g., IL-17-producing γδ T cells or IFN-γ-producing γδ T cells have been described [59]. There has been accumulating evidence demonstrating γδ T cells are involved in various aspects of the host immune responses ranging from acute and chronic inflammation to killing virus-infected cells, cancerous cells, and stressed cells [60, 61].

It has also been confirmed that both naturally IFN-γ- or IL-17-producing γδ T cell subsets express different NK cell receptors [including NK group 2 member D (NKG2D) receptors and killer immunoglobulin-like receptors (KIRs)] that are responsible for controlling the γδ T cell-mediated immune responses [62]. On the other hand, in order to terminate γδ T cell responses, several inhibitory receptors can exert negative signals, specifically programmed cell death-1 (PD-1) and B and T lymphocyte attenuator (BTLA) [62].

### γδ T cell-mediated innate and adaptive immune responses

γδ T cells are regarded to be innate-like lymphocytes with crucial role in immune responses or immune regulations by detecting antigens directly in a MHC-unrestricted priming manner, resulting in response to stress conditions rapidly without the need for clonal selection and differentiation [15]. In the initiation phase of immune responses, γδ T cells may connect innate and adaptive immunities, thereby playing crucial roles in anti-infection, autoimmunity, and antitumor effects [18, 19]. Moreover, they produce a large number of inflammatory molecules, such as granulocyte–macrophage colony-stimulating factor (GM-CSF), IFN-γ, and tumor necrosis factor (TNF) [63] and also can stimulate other immune cells including monocytes, neutrophils, dendritic cells, B lymphocytes, and different subtypes of T cells [64]. In this context, in humans, γδ T cells induce B cell class switching by expressing several factors including: (1) IL-4 and IL-10 cytokines, involved in immunoglobulin class switching; (2) CXCR5, a chemokine receptor localizing B cells in follicles; (3) CD40 ligand (CD40L), promoting B cell activation; (4) IL-21 and IFN-γ cytokines, which are crucial for the development of follicular helper T cells and germinal center-resident B cells, suggesting a direct role for γδ T cells in modulating humoral immunity by helping B lymphocytes to produce different classes of antibodies [65–67].

In comparison to αβ T cells, γδ T cells not only participate more extensively in innate immunity and homeostatic processes [68], but also actively produce IL-17 and IFN-γ in response to antigenic stimulation in tissues [69]. Several studies have shown that IL-17 demonstrates both protective and pathogenic features in the immune system and the involvement of this cytokine is of importance in host defense against various infectious organisms [70–72]. These non-conventional T cells can also promote adaptive-like responses by sharing functions with APCs, pro-inflammatory and cytotoxic effector cells, and immune-regulatory cells [73]. Consequently, before effective expansion and activation of antigen-specific αβ T cells, γδ T cells represent the first line of immune barrier to prevent infection from pathogens [74].

### γδ T cells role in antiviral immunity

Despite the role of NK cells and CD8^+^ αβ T cells in the early and the late phase of controlling viral infections (respectively), some studies have addressed the imperative role of T lymphocytes expressing the γδ T cell receptor in reducing viral loads by direct lysis of virus-infected cells and by non-cytolytic mechanisms, particularly γδ T cell-induced bystander activation of other immune cells, as summarized in Table 1.

**Table 1:** Antiviral activity of γδ T cell subsets in in various experimental models

Viral infection	Experimental model	γδ subsets	Antiviral mechanism	References
HCV	Co-cultures of Rep60 cells with either patients’ or healthy donors’ PBMC, or highly purified γδ T cells	Vγ9Vδ2	Vγ9Vδ2 T cell-mediated IFN-γ-dependent anti-HCV activity	[86]
HIV	Infection of PBMCs of healthy donors with HIV	Vγ9Vδ2	HIV replication inhibition as a result of releasing CCR5 ligand chemokines by activated Vγ9Vδ2 T cells	[91]
	PBMCs of healthy donors and HIV-1-infected patient, as well as HIV-1-infected PM1 cell line	Vδ1	NKp30-induced Vδ1 T cell-mediated HIV-1 replication inhibition through β chemokines production	[92]
	Co-culture of γδ T cell-enriched from PBMCs of HIV^+^ and healthy donors with HIV-infected CD4^+^ T cells	Vδ1^+^	A significant increase in Vδ1^+^ T cells expressing an activating NKG2C receptor in HIV-infected patients, compared with healthy donors NKG2C-induced Vδ1^+^ T cell-mediated cytotoxicity in response to HIV infected- CD4 T cells expressing NKG2C ligand A possible involvement of NKG2C-bearing Vδ1 T cells in reducing the number of CD4^+^ T cells observed in HIV^+^ persons with chronic untreated viraemia	[77]
			Vδ1^+^ T cell-mediated killing of infected cells in a perforin-dependent mechanism	[76]
	A single cell line p815 coated with rabbit antibodies specific to p815 cells, as target cells accompanied by PBMs isolated from HIV^+^ and HIV^−^ donors, as effector cells	Vγ2Vδ2	CD16-mediated ADCC by Vγ2Vδ2 T cells against antibody coated target cells The potential capacity of CD16^+^ Vδ2^+^ T cells as potent ADCC effector cells to control HIV-1 disease progression	[78]
EBV	Co-culture of Vγ9Vδ2 derived from seropositive EBV health donors’ PBMC with EBV-transformed autologous lymphoblastoid B cell lines (EBV-LCL) Inoculation of EBV-LCL to humanized Rag2^−/−^ γc^−/−^ mice	Vγ9Vδ2	Cytotoxic activity of Vγ9Vδ2 T cells against EBV-LCL based on NKG2D triggering, as well as Fas/FasL- and TRAIL-mediated apoptosis A possible involvement of IFN-γ in Vγ9Vδ2 T cell-mediated suppression of EBV-LCL proliferation in humanized mice The potential function of Vγ9Vδ2 T cells in the control of EBV-induced lymphoproliferative disease (EBV/LPD) by killing of EBV-LCL in humanized mice	[79]
SIV	Macaques model of SIV infection	Vδ1/Vδ2	NKG2D-, IFN-γ-, and granzyme B-mediated antiviral activity of γδ T cells	[93]
SARS-CoV	Co-culture of Vγ9Vδ2 T cell lines isolated from PBMC of patient and healthy donors with SARS-CoV-infected THP-1 cells, a human monocyte cell line	Vγ9Vδ2	Anti-SARS activity mediated by Vγ9Vδ2 T cells in an IFN-γ-dependent mechanism The possible engagement of NKG2D in triggering the cytolytic activity of Vγ9Vδ2 T cells against SARS-CoV-infected cells	[94]
ZIKV	Co-culture of ZIKV-infected A549 cells with PBMC of healthy donors	Vδ2^+^	NKG2D-mediated perforin release by Vδ2^+^ T cells in response to ZIKV-infected virus expressing NKG2D ligands	[80]
DV	Co-cultures of autologous DV-infected dendritic cells with either patients’ or healthy donors’ PBMC, PBL, or purified γδ T cells	pan γδ	γδ T cell-mediated rapid anti-DV activity by producing IFN-γ and up-regulating CD 107a as a marker of degranulation Monocyte-mediated enhancement of γδ T cell responses against DV-infected cells in an IL-18-dependent manner	[95]
HCMV	Incubation of γδ T cell lines isolated from KTRs or healthy donors with IgG opsonized HCMV-infected fibroblasts	Vδ2^neg^	Co-operation of HCMV-induced CD16^+^ Vδ2^neg^ T cells with anti-HCMV IgG recognizing infected cells to suppress virus propagation in an INF-γ-dependent mechanism Synergistic effects of IL-12 and IFN-α produced during HCMV infection on the enhancement of CD16-induced IFN-γ secretion	[90]
	Incubation of γδ T cell colons from patients’ or healthy donors’ PBMCs with models of HCMV infection in cell lines		HLA- and NKG2D-unrestricted TCR-mediated recognition and killing of CMV-infected cells by Vδ2^neg^ T cells in a perforin/granzyme B dependent pathway	[96]
MCMV	Normal and γδ T-cell-depleted mice	Vγ1^+^	The ability of IFN-γ-producing Vγ1^+^ T cells to respond quickly to HSP65 induced at early phase of MCMV infection A possible role of IL-12 and/or TNF-α produced during MCMV infection in increasing IFN-γ secretion by Vγ1^+^ T cells	[85]
WNV	Normal, αβ- and γδ-TCR deficient mice	pan-γδ	IFN-γ-mediated antiviral immunity against WNV infection provided by γδ T cells γδ T cell-induced perforin-mediated cytotoxicity	[75]
VV	Normal, αβ- and γδ- TCR deficient mice	pan-γδ	The critical function of γδ T cells in mediating innate immunity by rapid recruitment to the site of infection, as well as by rapid response to VV infection in an IFN-γ dependent mechanism	[83]
VSV	Normal and T cell deficient mice	pan-γδ	A possible role of γδ T cells as an alternative pathway in antibody isotype switching from VSV-specific IgM to IgG in αβ T cell-deficient mice via an IFN-γ-dependent manner	[82]
CNPV	Vaccination of human volunteers with live recombinant CNPV	Vγ9^+^	Expansion of CNP-specific IFN-γ-producing Vγ9^+^ T cells, as well as enhanced activity of NK cells (probably mediated by γδ T cells) in recipients of CNP vaccine A possible contribution of γδ T cell- and NK-produced IFN-γ to develop protective type-1 memory immunity during primary immune responses	[97]
HSV-1	The mouse model for corneal infection with HSV-1	pan-γδ	A dominant protective role for IFN-γ produced by γδ T cells, as well as macrophage-derived TNF-α and nitric oxide at early stages of HSV-1 infection A possible role of γδ T cells and macrophages as the early line of defense during acute HSV-1 proliferation in reducing the recurrence rate of herpetic disease	[84]
	HSV-induced cytotoxic activity of PBMCs from immune healthy donors and target cells	Vγ9Vδ2 (< 80%)	CD4^−^ CD8^−^ γδ T cell-mediated Killing of HSV-infected cells in an HLA-independent manner A possible involvement of accessory molecules like LFA-1 as an enhancer of TCR signaling in γδ T cell-mediated cytotoxic activity	[98]
CVB3	C57BL/6, BI.Tg.Eα, and γδ knockout mice	Vγ1^+^/Vγ4^+^	The contribution of γδ^+^ T cell subpopulations to host susceptibility to CVB3-induced myocarditis MHC class II antigen (IA and IE) restricted clonal selection of Vγ subpopulations during thymic development Promotion of CVB3-induced myocardial inflammation in BI.Tg.Eα [IA^−^IE^+^] mice by Vγ4^+^ T cells-mediated Th1-biased immunity T cell expressing Vγ1 gene-mediated suppression of CVB3-induced inflammatory damage in C57BL/6 [IA^+^IE^−^] mice by modulating Th2-biased immunity	[99]

HCV, Hepatitis C virus; Rep60, human hepatoma Huh7 cell line harboring HCV replicon; PBMCs, peripheral blood mononuclear cells; HIV, human immunodeficiency virus; MIP, macrophage inflammatory protein; RANTES, regulation on activation, normal T cell expressed and secreted; P815, mouse leukemia cell line; ADCC, antibody-dependent cellular cytotoxicity; EBV, Epstein–Bar virus; SARS-CoV, severe acute respiratory syndrome-associated coronavirus; ZIKV, Zika virus; DV, dengue virus; PBL, peripheral blood lymphocytes; HCMV, human cytomegalovirus*;* KTRs, kidney transplant recipients; HLA, human leukocyte antigen; MCMV, murine cytomegalovirus; HSP65, heat-shock protein 65; WNV, West Nile virus; VV, vaccinia virus; VSV, vesicular stomatitis virus; CNPV, canarypox virus; HSV-1, herpes simplex virus type 1; LFA-1, lymphocyte function-associated antigen-1; CVB3, coxsackievirus B3

It seems that γδ TCR-bearing T cells can exert their protective role in the killing of virus-infected cells directly through the perforin/granzyme cytotoxic pathway, the apoptosis pathways triggered by death inducible receptors like FAS and tumor necrosis factor-related apoptosis-inducing ligand receptors (TRAIL), the natural killer group 2, members C and D (NKG2C, D)-mediated γδ T cell recognition and destruction, and antibody-dependent cell-mediated cytotoxicity (ADCC) [9, 10, 75–78]. In West Nile virus (WNV) infected mice, a trend toward significantly lower perforin expression level was noted in TCRδ^−/−^ mice in comparison with wild type or TCRβ^−/−^ mice, suggesting the ability of γδ T cells to directly produce perforin or their potential to modulate the production of perforin by αβ T cells [75]. In human immunodeficiency virus (HIV)-positive patients, perforin-mediated HIV-infected CD4^+^ T cells destruction by Vδ1^+^ γδ T cells has also been reported [76]. Regarding Epstein–Barr virus (EBV)-infection, NKG2D receptor triggering and the cytotoxic pathways of TRAIL and Fas/Fas ligand (FasL) were outlined as the major mechanisms involved in clearing EBV-transformed autologous lymphoblastoid B cell lines by Vγ9Vδ2 T lymphocytes [79]. It has also been revealed that an up-regulation of NKG2D ligand expression on the surface of target cells induced by Zika virus (ZIKV) infection makes them more susceptible to lyse by NKG2D^+^ Vδ2^+^ T cells through perforin pathway [80]. Although NKG2D is originally known as an activating receptor for NK cells, it also acts as a potent co-stimulatory receptor of human Vγ9Vδ2 T cells. Furthermore, NKG2D-mediated cytotoxic function of Vγ9Vδ2 T cells by triggering the release of cytolytic granules through receptor-ligand recognition has been discussed recently [81]. Alternatively, NKG2C (an activating NK cell receptor of the C-type lectin)-mediated cytotoxicity was reported to be involved in the lysis of HIV-infected CD4^+^ T cells by NKG2C-bearing Vδ1^+^ γδ T cells [77]. Of importance, the potential role of Fcγ receptor III (CD16)-bearing Vδ2 T cells in the control of HIV type 1 disease through ADCC in the presence of specific antibodies that target antigens expressed on the surface of infected cells has been proposed [78].

The mechanisms underlying γδ T cell-mediated non-cytolytic antiviral activity consist of the production of cytokines (notably IFN-γ) and β chemokines like macrophage inflammatory protein (MIP)-1α, MIP-1β, and RANTES (regulation on activation, normal T cell expressed and secreted) [82, 83]. Upon infection with viruses like WNV, vaccinia virus (VV), hepatitis C virus (HCV), murine cytomegalovirus (MCMV), it has been reported that an early immune response (as a first line of host defense) involving IFN-γ-secreting γδ T cells is critical to control viral infection [75, 83–86]. IFN-γ presents potent antiviral activity by interfering with the viral replication through the induction of intracellular signaling pathways following interferon binding to its cell-surface receptors and subsequent up-regulation of a set of IFN-stimulated genes (ISGs) products that directly inhibit key steps of the replicative life cycle of viruses and by inducing the death of target cells through the modulation of both innate and adaptive immune responses [86–88]. However, little is known about the mechanism by which IFN-γ-secreting γδ T cells regulate the replication of different viruses.

After primary herpes simplex virus type 1 (HSV-1) infection in mice, it has been observed that viral replication is suppressed by IFN-γ-producing γδ T cells and macrophages that infiltrate into the infected trigeminal ganglion (TG). Cross-regulation between these two types of immune cells is required for their accumulation and antiviral functions in the TG. In this manner, synergistic effects of γδ T cell-produced IFN-γ with macrophage-derived TNF-α and nitric oxide (NO)-producing enzymes, i.e., inducible nitric oxide synthase (iNOs) can dramatically restrict viral replication [84, 89]. On the other hand, the possible contribution of IFN-γ-producing gamma-delta T cells to induce the neutralizing IgG responses independently of αβ T cell help in αβ T cell-deficient mice infected with either live vesicular stomatitis virus (VSV) or a recombinant vaccinia virus expressing the VSV glycoprotein has been described [82]. Of note, CD16 was also reported to be a TCR-independent activating receptor on human cytomegalovirus (HCMV)-induced Vdelta2-negative gamma-delta T cells, able to promote antiviral activity against IgG-opsonized HCMV-infected fibroblast cells by antibody-mediated CD16-induced IFN-γ secretion, called antibody-dependent cellular inhibition (ADCI) [90].

On the other hand, the production of antiviral factors MIP-1α, MIP-1β, and RANTES by Vγ9Vδ2-bearing lymphocytes has been shown to block HIV replication in vitro by inhibiting the CCR5 co-receptor that is required for HIV entry [91]. Alternatively, the involvement of NKp30, an activating receptor for NK cells, expressed on activated V delta 1 T cells in inducing β chemokines production, followed by the inhibition of CCR5-tropic HIV-1 strains replication has been described [92]. Taken together, unlike αβ T cells, γδ T cells, which possess innate and adaptive immune characteristics, play a crucial part in the front line of defense against various viral infections.

### Respiratory γδ T cells

Unlike αβ T cells, the preferential tropism of γδ T cells for non-lymphoid sites such as the skin, respiratory, digestive, and reproductive tracts may be related to their potential role in providing the early protection against invading pathogens and/or in mitigating the tissue-damaging side effects of immune responses in an immune regulatory manner [100, 101]. In this context, there is increasing evidence to reveal the role of γδ TCR-bearing T lymphocytes in maintaining and protecting normal functions of the airway, which has been reviewed in Ref. 100.

Heterogeneous populations of γδ T cells (predominantly Vγ1^+^ and Vγ4^+^) with distinct functions (mostly immunoregulatory role) are found in the lung [101, 102] and responses of lung-specific γδ T cells have also been reported in a variety of lung diseases including bacterial, viral, and fungal infections, allergic disease, inflammation and fibrosis, and cancer (reviewed in Ref. 101). For instance, during acute respiratory syncytial virus (RSV) infection, the beneficial role of activated γδ T cells as the main source of IL-17A in regulating viral responses and reducing lung inflammation in RSV-infected adult mice has been reported. Compared with adult mice, a significant decrease in γδ T cell-mediated IL-17A responses was noted in RSV-infected neonatal mice during initial infection. On the other hand, increased levels of IL-1β and IL-6 (as activated inflammasome markers) involved in the induction of IL-17A as a product of inflammasome-activated γδ T cells was also only observed in the lungs of RSV-infected adult mice, indicating a defect in inflammasome activation upon neonatal RSV infection. Interestingly, lung pathogenesis in neonates was reduced by exogenous IL-17A administration, while neutralization of IL-17A resulted in increased inflammatory responses in adult mice, suggesting the inflammation-inhibiting effects of IL-17-producing γδ T cells in the mouse model of RSV disease [103].

However, the increased recruitment of Vγ4^+^ γδ T cells to the lung and subsequent production of IFN-γ, TNF, RANTES, IL-10, IL-4, and IL-5 in a time-dependent manner (i.e.*,* γδ T cell-mediated early production of Th1, followed by a late surge of regulatory/Th2 cytokines release) were observed after RSV challenge in BALB/c mice immunized with a live vaccine vector expressing RSV F protein. During the secondary challenge, In vivo depletion of γδ T cells did not affect viral clearance but resulted in reduced lung inflammation and slightly increased peak viral replication in vaccinated mice [104]. Such differences as cytokine profile and/or γδ T cell-mediated disease pathology (e.g. alleviating or inducing lung inflammation) seems to be related to the presence of distinct populations of γδ^+^ T cells in the lung with specific roles [100].

### γδ T cell effects in defense against influenza infection

Recently, it has been observed that γδ T cells (mostly GV9-TCRγ-expressing γ9δ2) are able to destruct influenza A virus-infected cells as efficient as CD8^+^ T cells or NK cells in a polycytotoxic manner and by releasing IFN-γ against infected cells in vitro [61]. Investigation on pigs, as human-like IAV infection models, has substantiated the role of γδ T cells as early defending modules of the innate-like, adaptive immune system in anti-H1N1pdm09 infection activities. Significant augmentation of γδ T cells-induced perforin secretion in IAV-infected pigs’ nasal mucosa along with enhancement of γδ T cells-producing CD8 in respiratory system lymph nodes constitute ample evidence to indicate the effectiveness of γδ T cells in controlling IAV infection [105].

IL-17A-producing γδ T cells were shown to be involved in initiating protective immunity and mediating lung homeostasis and repair in a neonatal mouse model of influenza virus. During neonatal influenza (H3N2) virus infection, lung accumulation of IL-17A-producing γδ T cells, associated with the increase in lung epithelial cell-derived IL-33, resulted in IL-17A-dependent IL-33-mediated promotion of local type II immunity and infiltration of group 2 innate lymphoid cells and regulatory T cells to the lung, which thereby led to the augmented amphiregulin secretion and tissue repair [14]. A very recent study has revealed that immediately upon infection with influenza virus, Vγ4+ γδ T cells, infiltrated by means of CXCR3 receptor, undertake the task of providing early defense barrier against infection. This mission is followed by induction of IL-17A production, aimed at activation of protective neutrophils and NK cells in tracheal mucosa and so suppressing invasive viral replication after influenza infection [106]. Similarly, co-culturing H3N2-infected human nasal epithelium with PBMC showed that IAV infection stimulates both NK cells and γδ T cells (especially Vδ2 γδ T cells) against infected targets in vitro [107]. In myxovirus protein 1 (Mx1)-congenic C57BL/6 mice models, those consuming a low-carbohydrate, high fat ketogenic diet (KD) showed lower viral titers and improved survival in response to lethal IAV infection due to IL-17-producing γδ T cell-mediated killing of IAV-infected airway epithelial cells, expanded in response to KD in the lung, and KD-expanded γδ T cell-mediated improved lung barrier integrity [108].

In a mouse model of influenza (H3N2) virus infection, it was observed that in the lung of infected mice, the percentage of V gamma 4^+^ γδ T cells was gradually augmented post-infection, peaked at day 10, which was paralleled by an increase in the number of inflammatory macrophages expressing heat-shock protein (HSP) mRNA. However, V gamma 2^+^/V gamma 1^+^ γδ T cells were the dominant γδ T cell phenotype on day 13 after infection, indicating the possible role of some of these lymphocytes in responding to HSP^+^ cells and resolving inflammatory process upon influenza infection [13]. It was also found that an increased frequency of IL-22-producing retinoic acid receptor-related orphan receptor-γt (RORγt)-positive αβ and γδ T cells, and innate lymphoid cells in the initial phase of influenza A (H3N2) virus infection was associated with the limited lung inflammation and so a more controlled secondary bacterial infection. Of note, during lethal and sublethal viral infection, high levels of IL-22 had no effect on the control of virus propagation and the development of virus-specific CD8^+^ T cell response [109]. Moreover, IL-21 has been shown to have a key modulatory role in controlling γδ T cells propagation in the respiratory tract and its presence is necessary for preventing hyperinflammatory responses of γδ T cells through overexpression of IL-17 and subsequent lung injury [110]. Similarly, depletion of serpinb1a in IAV-infected mice models resulted in augmentation of IL-17^+^ γδ T cells and CD4^+^ Th17 cells and so increased the rate of mortality and impaired immune responses among depleted mice, mentioning the anti-inflammatory role of serpinb1a in IAV-driven overproduction of γδ T cells [111]. Nevertheless, in wild-type mice, the detrimental effect of type I IFNs induced during primary nonlethal influenza infection on increased susceptibility to secondary *Streptococcus pneumonia* infection by negative regulation of lung γδ T cell-mediated IL-17 production and IL-17-induced neutrophil recruitment, involving in lung immunity to bacterial infection, has been reported [112]. In the context of postinfluenza occurrence of lethal pneumococcal pneumonia, it has been shown that IL-27 expression in response to IAV infection hinders IL-17-expressing γδ T cells production by involvement of STAT1 factor and thereby deprives the host from the immunity related to IL-17 expression by γδ T cells after initial influenza infection [113].

The non-specific protective role of trehalose 6,6′-dimycolate (TDM) against influenza virus infection by inducing lung accumulation of γδ T cells following intravenous exposure in mice has been described. Depletion of γδ T cells in vivo impaired the resistance of TDM-treated mice to lethal influenza A (H3N2 and H1N1) virus infection, as did depletion of either αβ T cells or NK cells, indicating a complementary role for γδ T cells, αβ T cells and NK cells in protecting mice against influenza virus infection. On the other hand, γδ T cells from TDM-treated mice exhibited a significant role in the host defense against influenza virus infection by recognizing an unprocessed whole HA molecule of either H1 or H3 subtype in a major histocompatibility complex-independent manner [114].

Highly pathogenic avian influenza (HPAI) H5N1 viruses have recently been reported to activate γδ T cells as the first line of the host immune defense via the trimmers of viral HA proteins, leading to the up-regulation of CD69 as an early activation marker on γδ T cells. This activation depends on both sialic acid receptors on the surface of γδ T cells and the glycosylation of HA proteins. In this manner, the protective activity of HA-induced γδ T cells against HPAI H5N1 virus infection is mediated directly through IFN-γ production or indirectly through the regulation of other immune cells by secretion of immunomodulatory cytokines. Moreover, H5N1 infected TCRδ^−/−^ mice showed an increased mortality rate and more severe lung injury, revealing the important part played by γδ T cells in the infection control. On the other hand, the rapid response of γδ T cells to viral infection, as well as the resistance of γδ T cells to HPAI H5N1infection increase the ability of these lymphocytes to control viral infection over other immune cells [60]. On the contrary, after Vγ4^+^ γδ T cell subset depletion, the survival rate of mice infected with the influenza A (H1N1) pdm09 virus was improved by reducing Vγ4^+^ γδ T cell-mediated IL-17 secretion, lung immunopathology injury, and inflammatory cell infiltration [115]. These contradictory results are likely due to the presence of different γδ T cell subsets in the lung with distinct functions and/or due to the use of different influenza virus strains and treatment methods [60, 100]. In this context, it has also been demonstrated that CD56^+^ subset of Vγ9Vδ2 T cells show higher degree of cytotoxicity against IAV infection when compared to CD56^−^ equivalent, which is due to the specific CD16-mediated degranulation concomitant with CD56^+^ Vγ9Vδ2 T cells activity [116].

Using a model of A549 cells, human lung alveolar epithelial cells, infected by human (H1N1) influenza virus, it was found that aminobisphosphonate pamidronate (PAM)-expanded Vγ9Vδ2 T cells from PBMC of healthy donors were efficiently able to control influenza pathogenesis by non-cytolytic inhibition of influenza replication and by direct killing of influenza-infected cells through cytolytic pathways of perforin/granzyme B, TRAIL, and Fas-FasL in a NKG2D-mediated cell to cell contact-dependent manner [9]. Regarding the ability of the influenza virus to replicate in human macrophage, human Vγ9Vδ2 T cells that are activated by IPP in vitro have also been demonstrated to have potent cell-killing activity in human (H1N1) and avian (H9N2 or H5N1) influenza virus-infected human monocyte-derived macrophages (MDMs) as the model, suggesting the heterotypic nature of Vγ9Vδ2 T lymphocyte responses to influenza A subtypes. The cytotoxic activity of activated-Vγ9Vδ2 T cells requires NKG2D recognition and is mediated by both Fas-FasL and perforin/granzyme B pathways [10]. Furthermore, in an IAV-infected MDMs model, virus-activated Vγ9Vδ2 T cells driven from PBMs of healthy donors were shown to preferentially express the chemokine receptors CCR1, CCR5, and CXCR5, among which the CCR5 is associated with the regulation of Vγ9Vδ2 T cell trafficking to virus-infected cells by binding to its ligands (CCL3, CCL4, and CCL5) produced in response to influenza infection. Of note, compared to human (H1N1) influenza virus, considerably higher Vγ9Vδ2 T cell-mediated expression of CCR5 chemokine ligands is stimulated by avian (H5N1 and H9N2) influenza viruses, which may be due to higher severity of infections among avians [11].

In young children, the role of γδ TCR^+^ IFN-γ^+^ T cells induced by trivalent live-attenuated influenza vaccine (LAIV) in providing further protective effects against influenza infections, compared with trivalent inactivated influenza vaccine (TIV), has been proposed [117]. After influenza vaccination, in young recipients, virus-activated γδ T cells were significantly higher than those in elderly group, indicating the possible negative effects of age-induced changes of γδ T cells responses on vaccine efficacy [118]. Interestingly, the frequency of T cells carrying γδ TCR in non-smokers was significantly higher than smokers following LAIV vaccination, indicating inhibitory effects of smoking in LAIV-induced alterations in the percentage of γδ T cells [119]. Concerning the harsh reaction of smokers suffering lung disease to respiratory infections, an intriguing study explored the effect of cigarette smoke on recovery from IAV infection among mice models and revealed that smoke-enhanced IL-17 production in the lung of mice exposed to cigarette smoke hampers the efficient activation of γδ T cells and so significantly decreases the possibility of survival after infection [120]. Additionally, it has been reported that obesity is correlated with the low numbers of γδ T cells in the peripheral blood, as well as reduced homeostasis and antiviral functions of γδ T cells, leading to increase the susceptibility of obese patients to respiratory infections (notably influenza virus) and subsequently higher rate of mortality among them. Also, remnant γδ T cells in obese individuals majorly lack the IL-2Rα and so are unable to be primed efficiently [121].

**Fig. 1:**
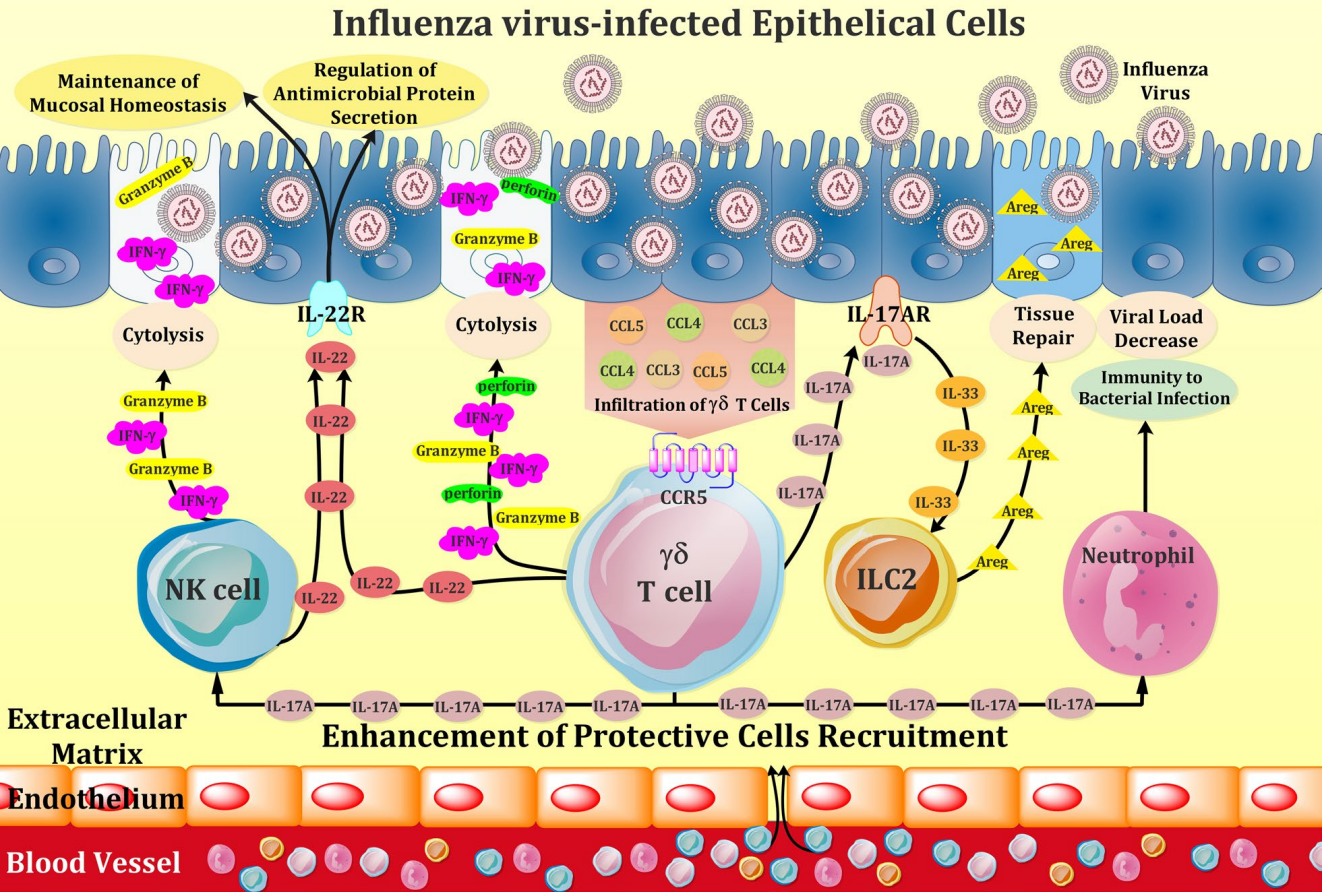
Schematic representation of effector mechanisms of γδ T cells in response to influenza virus infection of respiratory epithelium. Upon infection with influenza virus, infected cells secrete CCL3, CCL4, and CCL5 chemokines and thereby recruit γδ T cells, harnessing chemokine-binding CCR5 receptor, to the site of infection. Activated effector γδ T cells release cytokines and chemokines such as IFN-γ, IL-17, IL-22, as well as cytolytic proteins including perforin and granzyme B, which can directly lyse the infected cells and can also recruit other immune cells such as natural killer (NK) cells and neutrophils to aid killing or healing infected cells. Moreover, γδ T cells expressing IL-17A, binds to IL-17A receptor on lung epithelial cells, producing IL-33 and thereafter drive colonic group 2 innate lymphoid cell (ILC2) activation during influenza virus infection. The activated ILC2 produces Amphiregulin (Areg), which participates in pulmonary tissue repair upon influenza infection. In addition, IL-22, released from both γδ T cells and activated NK cells, is effective in preserving the homeostasis of mucosal barrier of influenza-infected respiratory tract and also plays a key regulatory role in microbial host defense after influenza infection

**Fig. 2:**
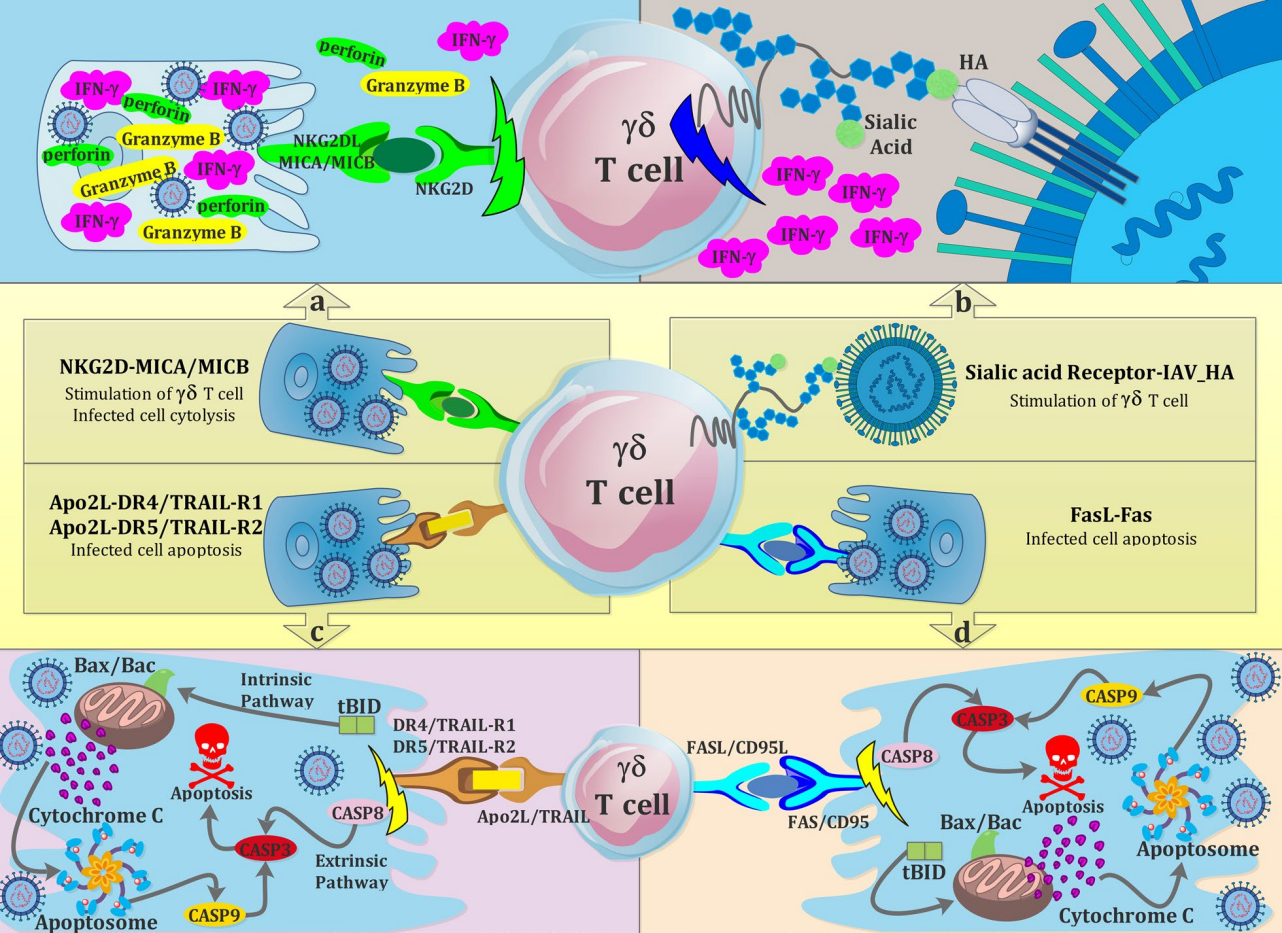
Cartoon overview of γδ T cells receptor-dependent interaction with influenza virus or influenza-virus-infected respiratory cells. **a** Activation of γδ T cells by the interaction between NKG2D (natural killer group 2D) receptor of γδ T cells with its ligands, MHC class I chain-related sequence A and B (MICA and MICB), expressed on the surface of influenza-infected epithelial cells, inducing the release of some cytokines and cytolytic proteins, somehow halting the influenza infection. **b** The interaction of influenza haemagglutinin (HA) on the surface of influenza viruses with sialic acid receptors plays a key role in the activation of γδ T cells, triggering the production of interferon-γ (IFN-γ). **c** γδ T cells utilize TNF-related apoptosis inducing ligand (TRAIL) for killing of influenza-infected cells. Infected epithelial cells express death receptors TRAIL-R1 or TRAIL-R2, identifying their ligand (Apo2 ligand) on the surface of influenza infection-activated γδ T cells and undergo apoptosis through Caspase-dependent pathways. d) FAS ligand expressed by γδ T cells, when exposed to FAS receptor available on influenza-infected cells surface activates the pathway of FAS-FASL apoptosis to destroy the infected cell

Regarding the possible correlation between variants of chromosome Y and incidence of IAV infection in males, it indicated that a number of particular ChrY consomic mice, like ChrY^PWD^ mice, are more susceptible to IAV infection. Further analysis attributed this phenomenon to higher levels of some IL-17-expressing subsets of γδ T cells (Vγ4^+^ and Vγ1^−^Vγ4^−^) in ChrY^PWD^ mice group without any observed alteration in level of IFN-γ among them, which together increase the fatality rate [122]. Collectively, these studies highlight a crucial role for γδ T cells in conferring protection and recovery from influenza virus infections. γδ T cell-mediated antiviral activities to eliminate influenza-virus-infected respiratory cells are shown in Figs. 1 and 2. Also, an overview of IAV-induced γδ T cell responses is summarized in Table 2.

**Table 2:** Anti-influenza mechanisms of γδ T cells in the respiratory tract

Study	Host	γδ subsets	Anti-influenza mechanism
Sant et al*.* [61]	Human γδ T cells along with target IAV-infected THP-1 cells	Vγ9Vδ2	Vγ9Vδ2 T cell-mediated IFN-γ-dependent anti-influenza activity Killing of target cells by Vγ9Vδ2 T cells with an equivalent efficiency to CD8^+^ T cells and NK cells
Schwaiger et al*.* [105]	Pigs, as human-like IAV infection models	pan γδ	Anti-IAV activity mediated by γδ T cells in a perforin-dependent cytotoxic pathway An increase in the percentage of CD8 expressing γδ T cells in route of viral entry
Xi-zhi et al*.* [14]	A neonatal mouse model	pan γδ	IL-17A-producing γδ T cell-mediated increased lung epithelial cell-derived IL-33 IL-33-mediated lung repair through the infiltration of group 2 innate lymphoid cells and regulatory T cells to the lung
Palomino et al*.* [106]	C57BL/6 mice	pan γδ	Recruitment of γδ T cells to the site of infection in a CXCR3 receptor-dependent fashion IL-17A-producing γδ T cell-mediated enhanced protective cells (neutrophils and NK cells) recruitment to the tracheal mucosa
Goldberg et al*.* [108]	Mx1-congenic C57BL/6 mice models	pan γδ	KD-expanded γδ T cell-mediated improved lung barrier integrity Viral clearance during lethal influenza infection in airway epithelium by γδ T cells expressing IL-17
Carding et al*.* [13]	C57B1/6 J mice	Vγ4^+^ Vγ2^+^/Vγ1^+^	A possible role of γδ T cells in responding to HSP^+^ cells and resolving inflammatory process upon influenza infection
Ivanov et al*.* [109]	*IL-22*^*−/−*^ and wild type mice	pan γδ	IL-22-producing γδ T cell- dependent limited lung inflammation and increased immunity to bacterial infection at early phase of IAV infection
Li et al*.* [9]	Human γδ T cells along with IAV-infected A549 cells	Vγ9Vδ2	NKG2D-induced Vγ9Vδ2 T cell-mediated cytotoxicity in perforin/granzyme B, TRAIL, and Fas-FasL dependent pathways
Qin et al*.* [11]	Human PBMC infected with IAV	pan γδ	CCR5-mediated regulation of Vγ9Vδ2 T cell trafficking to virus-infected site by binding to its ligands secreted from infected cells upon IAV infections
Dong et al*.* [60]	TCR-δ^−/−^ and wild type mice	pan γδ	Unprocessed whole HA molecule-restricted activation of γδ T cells IFN-γ-mediated antiviral immunity against IAV infection provided by HA-induced γδ T cells γδ T cell-induced bystander activation of other immune cells by secretion of immunomodulatory cytokines
Stervbo et al*.* [118]	Human donors	pan γδ	Detrimental effects of age and smoking on LAIV-induced γδ T cell responses
Horvath et al*.* [119]			
Moser et al*.* [110]	IL-21R KO mice, C57BL/6, and CD45.1 mice	pan γδ	*Modulatory role of IL-21 and* serpinb1a *in the control of negative effects* of overstimulation of IL-17-producing *γδ T cells* in the respiratory tract after IAV infection
Zhao et al*.* [111]	Wild type and *serpinB1*^*−/−*^ mice		

IAV, influenza A virus; THP-1 cells, a human monocyte cell line; NK cells, natural killer cells; MX1, myxovirus protein 1; KD, ketogenic diet; HSP, heat shock protein; A549 cells, human lung alveolar epithelial cells; HA, hemagglutinin; LAIV, live-attenuated influenza vaccine

### γδ T cell-based emerging therapeutic approaches

There is increasing interest to find alternative strategies aimed to offer a safe and accessible option for treating viral infections. In this context, stimulatory targeting of γδ T cells to activate and allow them to show their effector functions quickly, regardless of virus subtypes and the emergence of drug-resistance viral strains, can be considered as a promising strategy. For instance, in humanized mice, exogenous administration of PAM, a common treatment for osteoporosis and Paget’s disease, has been shown to decrease the disease severity and the number of death caused by H1N1 and H5N1 influenza viruses by enhancing human Vγ9Vδ2 T cell immunity indirectly through the induction of intracellular accumulation of IPP, a metabolite in the mevalonate pathway that selectively expands human Vγ9Vδ2 T cells, suggesting the potential application of PAM in human clinical trials [123]. The potential applicability of PAM-induced activation of human Vγ9Vδ2 T cells to control EBV-induced lymphoproliferative disease has also been reported [79]. Using a co-culture system containing Huh7 hepatoma cells carrying the subgenomic HCV replicon (Rep60 cells) and PBMC of healthy and HCV^+^ donors, it was also found that treatment with drugs containing non-peptidic antigens [zoledronic acid (ZOL) and bromohydrin pyrophosphate (BrHPP)] could influence HCV replication by expanding IFN-γ-producing Vγ9Vδ2 T cell population, suggesting the feasibility of using non-peptidic drugs as an innovative therapeutic approach in patients suffering from HCV [86].

On the other hand, Vδ2^+^ γδ T cells from HIV-infected individuals were demonstrated to respond strongly to ZOL plus IL-2, leading to rapid expansion of CD16-expressing Vδ2^+^ subset associated with enhanced ADCC-dependent CD16-mediated antiviral activity of Vδ2^+^ γδ T cells, suggesting the capacity of ZOL/IL-2-expanded Vδ2^+^ γδ T cell-based immunotherapy in HIV patients [124]. Modulation of CD16^+^ Vδ2^neg^ γδ T cell-mediated ADCI by exogenous administration of HCMV-specific IgGs may also provide a promising anti-human CMV strategy [90]. ZOL-mediated human Vδ2 cell targeting that are involved in the control of WNV infection in vitro by both cytotoxic and non-cytotoxic mechanisms may facilitate a novel immunotherapy strategy to the treatment of WNV infection [125].

Of note, adoptive immunotherapy using HIV-1-reactive CD4-negative γδ T cells, able to mediate antiviral activity, may be playing a critical role in the control of HIV-1 infection. However, the advancement of γδ T cell-based adoptive cellular immunotherapy for HIV-1 infection has been constrained by the inability to isolate or expand sufficient numbers of viable γδ T cells [126].

Concerning the construction of a universal vaccine against distinct subtypes of influenza A virus, Jameson et al. showed that human PBMC-derived Vγ9Vδ2 T cells induce CD25 and CD69 expression and also produce IFN-γ upon IAV infection, promisingly suggest the ability of Vγ9Vδ2 T cells in providing effective cross-reactive responses to IAV infection. In addition, they found that incorporation of mevastatin (hydroxymethylglutaryl-CoA reductase inhibitor) has a negative effect on γδ T cells activation, emphasizing the role of mevalonate pathway in Vγ9Vδ2 T cells efficiency [12]. Interestingly, it has been demonstrated that daily consuming of cranberry alleviates the symptoms of influenza due to inducing γδ T cells proliferation and so reducing the inflammatory cytokines profiles, an akin phenomenon previously observed about aged garlic extracts and L-theanine present in tea beverage [127–130].

In view of what has been previously discussed, it seems that γδ T cell-based immunotherapy may complement the traditional therapeutic regimen currently used for treating viral infections, particularly influenza A infection. Information on the potential applicability of γδ T cells to elicit improved therapeutic approaches is also increasing**.** In this manner, the strategy of targeting γδ T cell by the commercially available drugs containing non-peptidic antigens, which are currently used in humans, can potentially offer a safe and accessible option for treating viral infections [123]. Of note, one concern regarding the clinical application of phosphoantigen-induced γδ T cells is whether these cells can enrich at the site of infections, such as the lung, upon influenza infection. In this context, the data regarding the impact of chemokines produced by influenza-infected cells on γδ T cell trafficking to the virus-infected site may accelerate their potential for immunotherapy [10]. However, the limitations associated with preparing the adequate number of desired γδ T cells have restricted the development of cell-target immunotherapy aimed to boost γδ T cells to clear infectious agent. Therefore, there is a need for developing additional strategies to overcome this obstacle [126].

## Conclusions

While annual vaccination exerts protection against influenza infection, comprehensive perception of immune system schemes, especially those related to the innate immune system immediately after infection, when confronting influenza virus may help to develop more efficient vaccines or improved anti-infection immunotherapies, resulting in a decline in the threat of influenza infection to human health. In this context, γδ T cells have been observed to be primed and activated upon influenza infection of the respiratory tract and play a mediating role to join the innate immune responses to adaptive ones. However, the efficacy of γδ T cells-induced immunity is dependent on various factors including the predominant subset of γδ T cells at the infection site, the amount of recruited γδ T cells, the host immune system status, and even the genetics of infected patients. Here, we recapitulated and discussed the latest discoveries in γδ T cells’ way of action upon influenza virus infection, aiming at showcasing one of the few but highly potential subsets of innate T cells, which can be considered as a tool for developing novel and improved protective or therapeutic agents.

## Data Availability

Not applicable.

## Abbreviations

PRRs: Pattern recognition receptors
PAMPs: Pathogen-associated molecular patterns
RLRs: Retinoic acid-inducible gene-I-like receptors
NLRs: NOD-like receptors
TLR: Toll-like receptor
IFNs: Interferons
NK cells: Natural killer cells
γδ T cells: Gamma-delta T cells
IPP: Isopentenyl pyrophosphate
MHC: Major histocompatibility complex
ILCs: Innate lymphoid cells
Areg: Amphiregulin
DC: Dendritic cell
APC: Antigen-presenting cell
cDCs: Conventional dendritic cells
pDCs: Plasmacytoid dendritic cells
DLNs: Draining lymph nodes
IAV: Influenza A virus
MAIT cells: Mucosal-associated invariant T cells
NKT cells: Natural killer T cells
TCR: T cell receptor
MR1: Major histocompatibility complex, class I-related gene protein
αGC: α-Galactosylceramide
iNKT: Invariant natural killer T cells
HA: Hemagglutinin
NA: Neuraminidase
Tregs: Regulatory T cells
Th: T-helper
V: Variable
C: Constant
J: Joining
IELs: Intraepithelial lymphocytes
NKG2D: NK group 2 member D
KIRs: Killer immunoglobulin-like receptors
PD-1: Programmed cell death-1
BTLA: B and T lymphocyte attenuator
GM-CSF: Granulocyte–macrophage colony-stimulating factor
TNF: Tumor necrosis factor
CD40L: CD40 ligand
TRAIL: Tumor necrosis factor-related apoptosis-inducing ligand receptors
ADCC: Antibody-dependent cell-mediated cytotoxicity
HIV: Human immunodeficiency virus
WNV: West Nile virus
FasL: Fas ligand
EBV: Epstein–Barr virus
ZIKV: Zika virus
MIP: Macrophage inflammatory protein
RANTES: Regulation on activation, normal T cell expressed and secreted
VV: Vaccinia virus
HCV: Hepatitis C virus
MCMV: Murine cytomegalovirus
ISGs: IFN-stimulated genes
HSV-1: Herpes simplex virus type 1
TG: Trigeminal ganglion
NO: Nitric oxide
iNOs: Inducible nitric oxide synthase
VSV: Vesicular stomatitis virus
HCMV: Human cytomegalovirus
ADCI: Antibody-dependent cellular inhibition
RSV: Respiratory syncytial virus
Mx1: Myxovirus protein 1
KD: Ketogenic diet
HSP: Heat-shock protein
RORγt: Retinoic acid receptor-related orphan receptor-γt
TDM: Trehalose 6,6′-dimycolate
HPAI: Highly pathogenic avian influenza
PAM: Aminobisphosphonate pamidronate
MDMs: Monocyte-derived macrophages
LAIV: Live-attenuated influenza vaccine
TIV: Trivalent inactivated influenza vaccine
ZOL: Zoledronic acid
BrHPP: Bromohydrin pyrophosphate
